# Synthesis and Evaluation of [^18^F]AlF-NOTA-iPD-L1 as a Potential Theranostic Pair for [^177^Lu]Lu-DOTA-iPD-L1

**DOI:** 10.3390/pharmaceutics17070920

**Published:** 2025-07-16

**Authors:** Guillermina Ferro-Flores, Myrna Luna-Gutiérrez, Blanca Ocampo-García, Nallely Jiménez-Mancilla, Nancy Lara-Almazán, Rigoberto Oros-Pantoja, Clara Santos-Cuevas, Erika Azorín-Vega, Laura Meléndez-Alafort

**Affiliations:** 1 Department of Radioactive Materials, Instituto Nacional de Investigaciones Nucleares (ININ), Ocoyoacac 52750, Mexico; guillermina.ferro@inin.gob.mx (G.F.-F.); myrna.luna@inin.gob.mx (M.L.-G.); blanca.ocampo@inin.gob.mx (B.O.-G.);; 2 Investigadora por México SECIHTI, Instituto Nacional de Investigaciones Nucleares, Ocoyoacac 52750, Mexico; nallely.jimenez@inin.gob.mx; 3 Department of Nuclear Forensics and Analytical Chemistry, Instituto Nacional de Investigaciones Nucleares, Ocoyoacac 52750, Mexico; nancy.lara@inin.gob.mx; 4 Faculty of Medicine, Universidad Autónoma del Estado de México, Toluca 50180, Mexico; rorosp@uaemex.mx; 5 Immunology and Molecular Oncology Diagnostics Unit, Veneto Institute of Oncology IOV—IRCCS, 35128 Padua, Italy

**Keywords:** PD-L1, PD-L1 peptide inhibitors, ^18^F-iPD-L1, iPD-L1 PET radiotracer

## Abstract

**Background/Objective**: Programmed cell death ligand-1 (PD-L1), which is overexpressed in certain tumors, inhibits the body’s natural immune response by providing an “off” signal that enables cancer cells to evade the immune system. It has been demonstrated that [^177^Lu]Lu-DOTA-iPD-L1 (PD-L1 inhibitor cyclic peptide) promotes immune responses. This study aimed to synthesize and evaluate [^18^F]AlF-NOTA-iPD-L1 as a novel radiotracer for PD-L1 positron emission tomography (PET) imaging and as a potential theranostic pair for [^177^Lu]Lu-DOTA-iPD-L1. **Methods**: The NOTA-iPD-L1 peptide conjugate was synthesized and characterized by U.V.-vis, I.R.-FT, and UPLC-mass spectroscopies. Radiolabeling was performed using [^18^F]AlF as the precursor, and the radiochemical purity (HPLC), partition coefficient, and serum stability were assessed. Cellular uptake and internalization (in 4T1 triple-negative breast cancer cells), binding competition, immunofluorescence, and Western blot assays were applied for the radiotracer in vitro characterization. Biodistribution in mice bearing 4T1 tumors was performed, and molecular imaging (Cerenkov images) of [^18^F]AlF-NOTA-iPD-L1 and [^177^Lu]Lu-DOTA-iPD-L1 in the same mouse was obtained. **Results**: [^18^F]AlF-NOTA-iPD-L1 was prepared with a radiochemical purity greater than 97%, and it demonstrated high in vitro and in vivo stability, as well as specific recognition by the PD-L1 protein (IC_50_ = 9.27 ± 2.69 nM). Biodistribution studies indicated a tumor uptake of 6.4% ± 0.9% ID/g at 1-hour post-administration, and Cerenkov images showed a high tumor uptake of both [^18^F]AlF-NOTA-iPD-L1 and ^177^Lu-iPD-L1 in the same mouse. **Conclusions**: These results warrant further studies to evaluate the clinical usefulness of [^18^F]AlF-NOTA-iPD-L1/[^177^Lu]Lu-DOTA-iPD-L1 as a radiotheranostic pair in combination with anti-PD-L1/PD1 immunotherapy.

## 1. Introduction

Programmed cell death ligand-1 (PD-L1) is a protein found in some normal cells but is highly expressed in several types of tumor cells and cancer-associated fibroblasts (CAFs) [1]. Its function in the tumor microenvironment is to bind to the immune checkpoint protein programmed cell death-1 (PD-1), which is expressed on regulatory T cells, thereby inhibiting immunogenic effects by providing an “off” signal that allows cancer cells to evade the immune system [2]. CAFs induce cancer cells to express higher levels of PD-L1. Patients with more PD-L1 have a poorer prognosis due to inactivation and suppression of the immune response. For example, in esophageal cancer, patients with CAFs that are positive for PD-L1 have worse survival than those with CAFs that are negative for PD-L1 [3].

Immune checkpoint inhibition (ICI) therapies, including PD-1/PD-L1-targeted immunotherapies, have demonstrated significant clinical benefits in the treatment of lung cancer and triple-negative breast cancer by enhancing antitumor immune responses through the blockade of PD-1/PD-L1 binding, thereby inhibiting their immunosuppressive function [4,5].

Radiation therapy is helpful in the treatment of many solid tumors. Radiation damages tumor cell DNA and alters the tumor microenvironment [6]. This is achieved by releasing tumor antigens, thereby making tumor cells more immunogenic, promoting inflammatory stress, activating immune cells, and ultimately activating the antitumor immune response [7]. However, radiotherapy also induces T cells and tumor cells to produce more PD-1 and PD-L1, respectively, which can potentially stop the immune response and cause tumors to acquire a radioresistant phenotype [8,9,10]. The objective of combining radiotherapy with anti-PD-1/anti-PD-L1 antibodies is to enhance the efficacy of radiotherapy while mitigating the unwanted immunosuppressive effects [11].

Recently, we proposed the use of a novel ^177^Lu-labeled PD-L1 peptide inhibitor ([^177^Lu]Lu-DOTA-iPD-L1) as a radiopharmaceutical with immunomodulatory properties, which, when combined with immunotherapy, may represent an improved approach to treating patients with tumors that express PD-L1 [12].

As a reproducible marker, PD-L1 is useful for monitoring response and guiding immunotherapy decisions in cancer patients. However, in addition to inter- and intra-neoplastic heterogeneity, PD-L1 expression is highly dynamic and changes over time. Therefore, SPECT and PET images showing PD-L1 expression heterogeneity within tumors are valuable for diagnosing PD-L1 positivity with high precision in selecting patients to receive anti-PD1, anti-PD-L1, and/or [^177^Lu]Lu-DOTA-iPD-L1 therapy.

In this context, the aim of this research was to synthesize and evaluate, in vitro and in vivo, [^18^F]AlF-NOTA-iPD-L1 as a novel radiotracer for PD-L1 PET imaging, which could potentially be used as a theranostic pair of [^177^Lu]Lu-DOTA-iPD-L1.

## 2. Materials and Methods

### 2.1. Molecular Modeling

**Figure A1:**
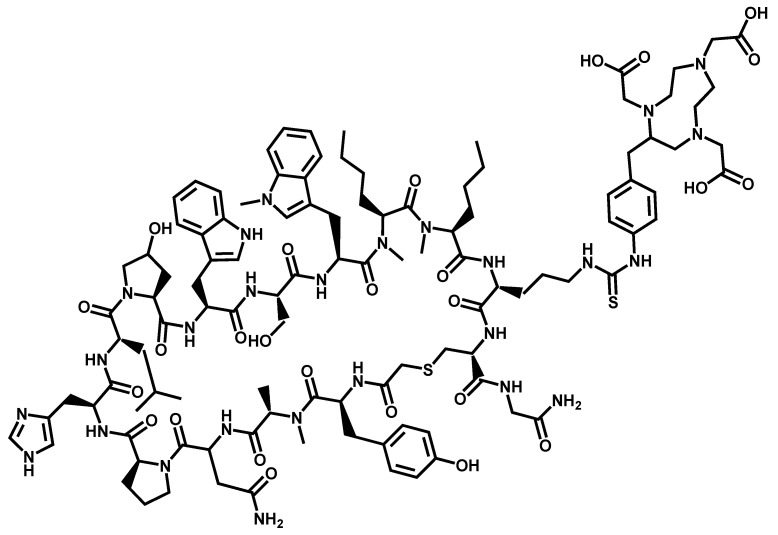
Schematic chemical structure of the NOTA-benzene-SCN-W12 (NOTA-WL12) ligand.

**Figure A2:**
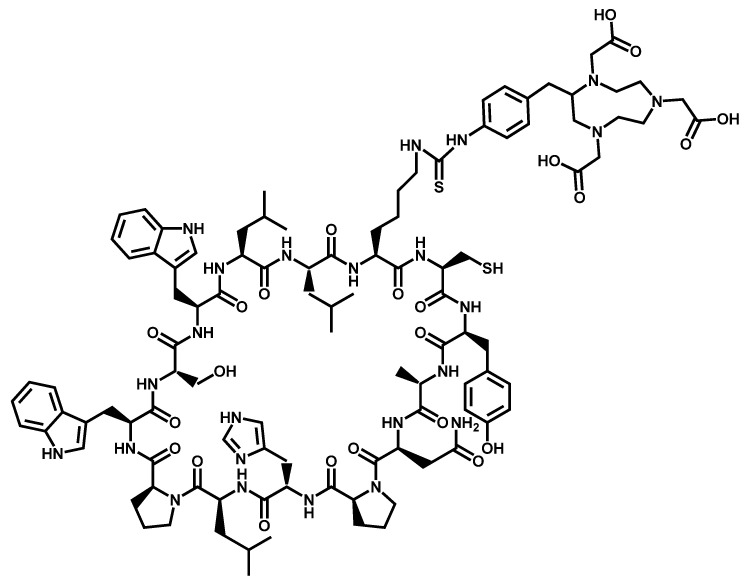
Schematic chemical structure of the NOTA-benzene-SCN-iPD-L1 (NOTA-iPD-L1) ligand.

The NOTA-WL12 (Figure A1) and NOTA-iPD-L1 (Figure A2) structures were created in 3D-pdb and pre-optimized with MMFF94 molecular mechanics, followed by PM7 quantum mechanics optimization (Mopac2016). For molecular docking, the receptor (human programmed death-1 ligand, PDB ID 4ZQK) and the PD-L1 inhibitor peptides (NOTA-iPD-L1 and NOTA-WL12) were prepared in a cubic geometry with 80 Å at the receptor center using AutoDock 1.5.7. Molecular docking was performed using AutoDock Vina (version 1.1.2), generating twenty poses for each inhibitor. The inhibition constants (Ki) were calculated based on affinity, temperature, and the universal gas constant (R), as previously reported [12].

### 2.2. Preparation and Characterization of NOTA-iPD-L1

WL12 is a programmed death ligand-1 (PD-L1) inhibitor peptide consisting of 14 amino acids (4 of which are methylated) that are linked by an -S- bond (https://patents.google.com/patent/US9879046B2/en, accessed on 1 June 2025). In 2024, our group developed the cyclic peptide iPD-L1 labeled with ^177^Lu, which does not contain an -S- bond or methylated amino acids. [^177^Lu]Lu-DOTA-iPD-L1 demonstrated higher uptake and internalization in PD-L1-positive cancer cells than [^177^Lu]Lu-DOTA-WL12 [12]. In this study, NOTA-iPD-L1 was prepared by conjugating the macrocycle 2-S-(4-isothiocyanatobenzyl)-1,4,7-triazacyclononane-1,4,7-triacetic acid (NOTA-benzene-SCN) to the amino terminal group of lysine in iPD-L1. Briefly, 2.1 µmol (4.0 mg) of iPD-L1 and 2.5 µmol (1.4 mg) of NOTA-benzene-SCN in 1.0 mL of DMF containing 40 µg of DIPEA were reacted under stirring for 16 h at room temperature. Next, the solution was purified by dialysis (dialysis tubing with a molecular weight cutoff of 1 kDa) against injectable-grade water, followed by lyophilization. It is important to note that adding DIPEA to maintain a basic medium significantly reduces the formation of a dithiocarbamate with cysteine in iPD-L1. As is well known, a basic medium favors the reaction of primary amines with isothiocyanate, whereas a neutral pH favors a click reaction between the -SH groups and isothiocyanate.

The NOTA-iPD-L1 conjugate was chemically characterized using FT-IR spectroscopy (from 400 to 4000 cm^−1^; ATR; PerkinElmer, MA, USA), UV-Vis spectroscopy (from 200 to 400 nm; LambdaBio; PerkinElmer, MA, USA), and UPLC with a QDa mass detector (Waters, Milford, CT, USA). For comparative purposes, NOTA-WL12 was also synthesized. UPLC with a QDa mass detector (Waters, Milford, CT, USA) was used for characterization. NOTA-WL12 was also synthesized by conjugating NOTA to ornithine for comparative purposes.

For binding assays, AlCl-NOTA-iPD-L1 was prepared by adding 150 µL of 2 mM AlCl_3_, dissolved in 1 M sodium acetate (pH 5), to 250 µg (250 µL) of the NOTA-iPD-L1 peptide dissolved in a 0.2:0.8 solution of 1 M sodium acetate (pH 5)/ethanol. The mixture was heated for 15 min at 100 °C and diluted to 1 mL with injectable water, followed by Sep-Pak purification.

### 2.3. Radioactive Labeling

Fluorine-18 was prepared as [^18^F]AlF (1110 MBq; 300 µL of 0.1 M sodium acetate, pH 4.1) at the cyclotron unit of the faculty of medicine at the National Autonomous University of Mexico (UNAM, Mexico City). NOTA-iPD-L1 or NOTA-WL12 (1 mg/mL) was dissolved in a 0.2:0.8 solution of 1 M sodium acetate (pH 5)/ethanol and sterilized by filtration (0.22 µm). Then, 250 µL of the peptide solution (250 µg of peptide) was mixed with 555 MBq (150 µL) of [^18^F]AlF and incubated at 100 °C for 15 min. The solution was diluted to 1 mL with injectable water, then purified using SepPak C18 cartridges (Waters, MA, USA) according to established protocols as follows: (1) Activation of the SepPak C18 cartridge with 5 mL of ethanol followed by 5 mL of injectable grade water, (2) Sample loaded into the cartridge (reaction solution), and (3) Cartridge elution with 5 mL of injectable grade water followed by the radiopeptide elution with an injectable grade water: ethanol 1:1 solution. This procedure yielded ^18^F-radiolabeled peptides in 2.0 mL of a 1:1 H_2_O:EtOH solution, with an overall radiochemical labeling yield of 29 ± 3% (*n* = 3). Cold peptide (10 mM) was added to half of each radiopeptide solution (1.0 mL) for subsequent in vitro and in vivo blocking studies. [^177^Lu]Lu-DOTA-iPD-L1 was also prepared for comparative imaging studies, as previously reported [12].

### 2.4. Radiochemical Purity Assessment

The [^18^F]AlF-NOTA-iPD-L1 and [^18^F]AlF-NOTA-WL12 chromatographic profiles for radiochemical purity evaluation were obtained using a Shimadzu chromatograph (Model LC-40 with UV detector and LB500 radioactivity detector) with a C18 column (Epic C18 column: L 250 mm, 5 µm particle size, and 4.6 mm internal diameter; PerkinElmer, MA, USA). The mobile phases were run in a linear gradient from 100% to 30% aqueous phase (H_2_O/CF_3_COOH 0.1% and CH_3_CN/CF_3_COOH 0.1%) at a flow rate of 1 mL/min. The retention times were 13.7 ± 0.2 and 14.3 ± 0.2 min for [^18^F]AlF-NOTA-iPD-L1 and [^18^F]AlF-NOTA-WL12, respectively. The retention time for [^18^F]AlF was 3.7–4.5 min. For comparative imaging studies, [^177^Lu]Lu-DOTA-iPD-L1 was also analyzed by HPLC (retention time of 14.0 ± 0.2 min), yielding radiochemical purities of 98.9 ± 1.1%, as previously reported in detail [12].

### 2.5. Serum Stability

To assess in vitro stability, 100 µL (20 MBq) of [^18^F]AlF-NOTA-iPD-L1 or [^18^F]AlF-NOTA-WL12 was introduced to 1 mL of human serum (Sigma-Aldrich, NIST-909c human serum certified sample; St. Louis, MO, USA). The mixture was incubated at 37 °C for 6 h, after which 0.5 mL of acetonitrile was added to precipitate the proteins. The mixture was then centrifuged at 500× *g* for 10 min. The radiochemical purity of the supernatant was analyzed by reverse-phase radio-HPLC, as described above (*n* = 3).

### 2.6. Partition Coefficient

To evaluate the partition coefficient, 0.05 mL (10 MBq) of either [^18^F]AlF-NOTA-iPD-L1 or [^18^F]AlF-NOTA-WL12 was added to 4 mL of a 1:1 mixture of water and *n*-octanol. The mixture was then centrifuged at 500× *g* for 5 min. One hour later, when the two solvent phases were clearly separated, 50 μL was taken from each layer. This sample was analyzed using a NaI(Tl) detector (γ-counter) to determine the partition coefficient values (partition coefficient = [cpm in n-octanol]/[cpm in water]) (*n* = 3). Finally, the compound’s lipophilicity was expressed as Log D.

### 2.7. Cell Culture

The HCT116 (CCL-247; human colorectal), 4T1 (CRL-2539; murine triple-negative breast cancer) and AR42J (CRL-1492; murine pancreatic) cell lines, obtained from the American Type Culture Collection (ATCC, Gaithersburg, MD, USA), were cultured in RPMI-1640 medium supplemented with fetal bovine serum and an antibiotic/antimycotic solution. Trypsin at 0.25% in PBS was used to detach the cells. Cell viability of greater than 85% was verified with trypan blue. Cells suspended in RPMI were incubated with specific treatments before being washed with PBS to determine the biological effects.

### 2.8. Affinity Assay

The competitive assay assessed the binding affinity of WL12 and AlCl-NOTA-iPD-L1 to the PD-L1 protein, which is highly expressed (+++) in 4T1 cells [12]. The cells, which had been adhered to 96-well plates, were incubated at 37 °C for one hour with different concentrations of unlabeled WL12 and AlCl-NOTA-iPD-L1 peptides (dilutions ranging from 10 to 0.00001 mM) (*n* = 3). The concentrations of [^18^F]AlF-NOTA-iPD-L1 were kept constant at 1 ng/20 kBq. The plates were then washed with a pH 7.4 buffer solution containing 0.1% BSA, 25 mM Tris-HCl, 1 mM CaCl_2_, and 120 mM NaCl, and the activity (% binding) was measured using a NaI(Tl) detector. The binding percentages were adjusted using non-specific binding values from an independent assay with [^18^F]AlF-NOTA (1 ng/20 kBq). Using GraphPad Prism version 10.5.0.774, we fitted the percentage capture as a competition curve to obtain the IC_50_.

### 2.9. Cell Internalization and Uptake

Tumor PD-L1 expression exhibits significant heterogeneity. Therefore, three cell lines were selected to assess the correlation between the uptake and internalization of [^18^F]AlF-NOTA-iPD-L1 and PD-L1 levels in cells with varying PD-L1 protein expression: high (4T1 cells), medium (HCT116 cells), and low (AR42J cells), as documented in previous studies [12]. Tubes containing 1 × 10^5^ cells (HCT116, 4T1, or AR42J) were prepared (*n* = 6). The blocked cells were exposed to non-radiolabeled PD-L1 inhibitor peptides (25 µg at a concentration of 1 µg/µL) for one hour. Then, radiolabeled peptides, [^18^F]AlF-NOTA-iPD-L1 or [^18^F]AlF-NOTA-WL12 (370 kBq), were added. After applying the ^18^F treatments to both blocked and unblocked cells, the tubes were incubated at room temperature for one hour. After incubation, the radioactivity in the cell suspension was measured (initial activity). Excess treatment was removed by centrifugation at 500× *g* for five minutes. The residual activity in the cell pellet was then resuspended in one milliliter of phosphate-buffered saline (PBS) to measure radioactivity (cell uptake fraction). The cells were then centrifuged again, and the pellet was resuspended in 1 mL of a 0.2 M acetic acid solution in 0.5 M NaCl solution. Another centrifugation and decanting followed this. Finally, the internalized fraction was evaluated by measuring the radioactivity of the cell pellet. Radioactivity measurements were performed in triplicate using a well counter with a NaI(Tl) detector. The percentage uptake was calculated by considering the initial activity to be 100%.

### 2.10. Immunofluorescence Tests

In immunodetection chambers (Nunc Merck, Darmstadt, Germany), 1.5 × 10^4^ cells (HCT116, AR42J, and 4T1) were cultured for 24 h under standard conditions. After adhering, the cells were fixed with a 4% paraformaldehyde solution in PBS for 30 min. Then, they were washed and blocked with a 2% BSA solution containing 0.5% Triton X-100 for 30 min. Next, the cells were incubated overnight at 4 °C with 3 µg/mL of a BSA-free anti-PD-L1 antibody (Novus Biologicals, Wuhan, China). To localize proteins, goat anti-mouse secondary antibody conjugated with Alexa Fluor 488 (A32731, Invitrogen, Thermo Fisher Scientific, Waltham, MA, USA) was used at a dilution of 1:1000 and incubated for 1 h at room temperature. The slides were allowed to dry and then mounted using Vectashield with DAPI (Vector Lab, Newark, CA, USA). The cells were viewed with a 40× objective on an MT-6200 microscope (Meiji, Japan) to verify PD-L1 expression and subcellular localization.

### 2.11. Western Blot Tests

**Figure A3:**
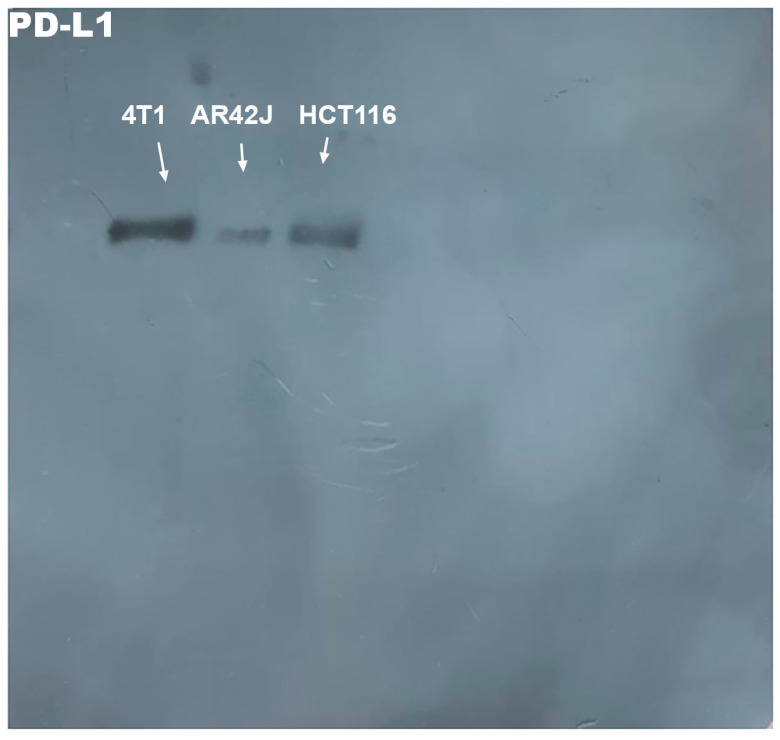
Original full Western blot gel (developing on a X-ray film) showing the PD-L1 expression in 4T1, AR42J, and HCT116 cancer cells.

First, the protein concentration in lysates from semi-confluent cultures was determined using a microplate spectrophotometer (Epoch, Thermo Scientific, Waltham, MA, USA). The samples were mixed at a 1:1 ratio with 2× Laemmli sample buffer (Bio-Rad, Hercules, CA, USA) and incubated at 95 °C for 5 min before being loaded onto a 4–15% polyacrylamide gel (Bio-Rad) in a Mini-Protean II run chamber (Bio-Rad). The gel was run at 100–80 V for approximately two hours. A 10–250 kDa protein ladder (Bio-Rad) was used as a reference. The proteins were then transferred to a 0.45 µm PVDF membrane (Merck Millipore, Darmstadt, Germania) at 100 V for one hour and washed with PBS-Tween (Calbiochem Biosciences, San Diego, CA, USA). The membrane was blocked with 4% BSA in PBST and incubated overnight at 4 °C with an anti-PD-L1 antibody (Abcam, Cambridge, UK), followed by incubation with goat anti-mouse IgG H&L conjugated to peroxidase. A chemiluminescent HRP substrate (Millipore, Burlington, MA, USA) was used for visualization, and bands were developed using X-ray film (Carestream Health, Rochester, NY, USA) (Figure A3). The membrane was then washed and stripped with a glycine/SDS/Tween solution. Finally, it was re-incubated with an anti-β-actin antibody (Sigma-Aldrich, St. Louis, MO, USA) as a loading control.

### 2.12. Biodistribution

The induction of tumors in 8-week-old female Balb-c mice was achieved by injecting 1 × 10^6^ 4T1 cells into the mammary gland. The mice were treated according to the established protocol No. CICUAL-01-25, which the ININ Internal Committee of the Care of Laboratory Animals had approved under the Mexican official standard (NOM-062-ZOO-1999; Technical Specifications for the Production, Care, and Use of Laboratory Animals. Publisher: Government of Mexico, Mexico City, Mexico, 2001). When tumors reach an average size of 0.5 cm in diameter, they are injected intravenously (tail vein) with 3.7 MBq/50 µL of [^18^F]AlF-NOTA-iPD-L1 and sacrificed at 1, 2, and 3 h (*n* = 3) after radiopharmaceutical administration. Subsequently, the excised organs or tissues (i.e., tumor, intestine, liver, spleen, lung, kidney, and heart) were removed, and the radioactivity uptake was quantified using a thallium-activated sodium iodide detector. The percentage of activity in each organ was calculated based on the initial activity injected into each mouse (aliquots of a reference standard), representing 100% of the activity. In the initial phase of the experiment, a group of mice (*n* = 3) was inoculated with 50 µL (2 mg/mL) of non-radiolabeled iPD-L1. Thirty minutes later, the mice were injected with 3.7 MBq/50 µL of [^18^F]AlF-NOTA-iPD-L1. Subsequently, the mice were euthanized one hour after radiopharmaceutical administration, and the percentage of radioactivity in each organ was evaluated as previously outlined. The %ID/tumor of this latter group was designated as the blocked receptor tumor group to ascertain whether the uptake in the tumors of the unblocked mice was specific.

### 2.13. Molecular Imaging

Luminescence images (Cerenkov imaging) of [^18^F]AlF-NOTA-iPD-L1 at one hour and ^177^Lu-iPD-L1 at 24 h post-administration were captured in mice (both radiopharmaceuticals in the same animal; ^177^Lu-iPD-L1 was injected 3 h after [^18^F]AlF-NOTA-iPD-L1 imaging) (*n* = 3) by using an imaging preclinical system (Preclinical Optical/X-ray/radioisotopic imaging system XTREME; Bruker, USA. One group of mice (*n* = 3) received 50 µL (2 mg/mL) of non-radiolabeled iPD-L1 peptide to block PD-L1 receptors, followed by 3.7 MBq/50 µL of [^18^F]AlF-NOTA-iPD-L1 after 30 min, and luminescent images were captured one hour after radiopharmaceutical administration. Furthermore, X-ray images were obtained from the entire bodies of the mice. All images were acquired with animals anesthetized with oxygen and 2% isoflurane in the supine position.

## 3. Results

### 3.1. Molecular Modeling

**Figure 1:**
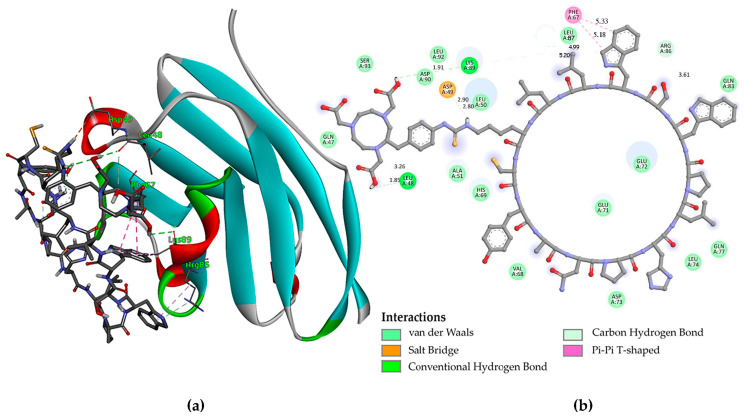
(**a**) The molecular docking study of the interaction between NOTA-iPD-L1 and PD-L1 indicated an affinity score of −6.3 kcal/mol and a Ki of 24.08 µM; (**b**) The primary chemical interactions of NOTA-iPD-L1 with selected amino acids of the protein are highlighted, with distances displayed on the Å scale.

**Figure 2:**
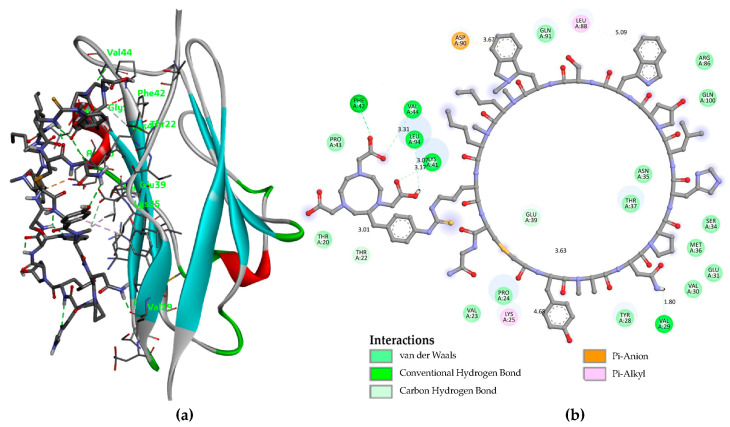
(**a**) The molecular docking study of the interaction between NOTA-WL12 and PD-L1 indicated an affinity score of −5.6 kcal/mol and a Ki of 78.48 µM; (**b**) The primary chemical interactions of NOTA-WL12 with selected amino acids of the protein are highlighted, with distances displayed on the Å scale.

Molecular docking calculations were performed using the PD-L1 receptor for the molecules NOTA-iPD-L1 and NOTA-WL12, the latter serving as a positive control. The results showed Ki values of 24.08 µM and 78.48 µM, respectively. The binding energies of the docked compounds were −6.3 kcal/mol and −5.6 kcal/mol for NOTA-iPD-L1 and NOTA-WL12, respectively. Figure 1 and Figure 2 show the types of bonds between the inhibitory peptides used in this study and the central amino acids of the PD-L1 protein. The docking results indicated that the NOTA-iPD-L1-derived peptide has a higher affinity than the NOTA-WL12 peptide. This difference may be due to a salt bridge formed by the Asp-49 residue in the PD-L1 protein and the hydrogen of the -N-(C=S)-(NH)- group (2.90 Å), which is the site where the NOTA–benzene–isothiocyanate molecule binds to lysine in the iPD-L1 peptide. It is important to note that although the NOTA-iPD-L1 and NOTA-WL12 peptides share several amino acids within the cyclic peptide, the significant difference in the amino acids of the protein’s active center that interact with the peptides is primarily due to the more restricted size of the iPD-L1 peptide ring conformation; this is because iPD-L1 lacks the -S- bond present in WL12.

### 3.2. Chemical Characterization

**Figure 3:**
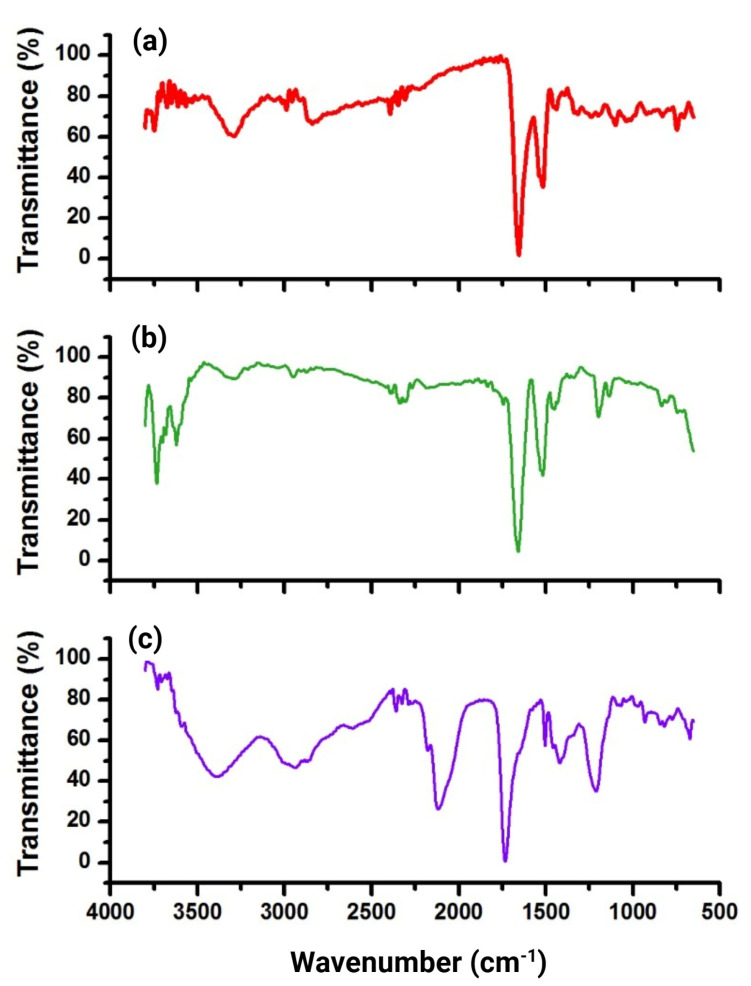
Fourier transform infrared spectra of (**a**) NOTA-iPD-L1, (**b**) iPD-L1, and (**c**) NOTA–benzene–SCN.

The FT-IR analysis was used to determine the formation of the binding thiourea and the conjugation reaction (Figure 3). The NOTA–benzene–SCN showed the S-C-N characteristic band at 2117 cm^−1^, which was not more pronounced in NOTA–benzene–SCN-iPD-L1, demonstrating the successful synthesis of NOTA-iPD-L1. The peaks attributed to the C=O bond of the peptide, originally located at 1660 cm^−1^ and 1450 cm^−1^ in the iPD-L1 spectrum (Figure 3b), were shifted to lower energies (1634 cm^−1^ and 1438 cm^−1^) in NOTA-iPD-L1 (Figure 3a).

In addition, the primary amine peak centered at 3299 cm^−1^ in the iPD-L1 spectrum shifted to lower energy at 3288 and 3291 cm^−1^, suggesting the interaction between this amine and the isothiocyanate motif of NOTA–benzene–SCN. Peaks located at 2883 cm^−1^ and 2981 cm^−1^, originally found in iPD-L1 and associated with the -CH_2_- bond, were shifted to 2869 cm^−1^, 2927 cm^−1^ and 2964 cm^−1^ in NOTA-iPD-L1. The δ vibrations of the N-H group at 1455 and 1436 cm^−1^ from iPD-L1 were modified in the new structure NOTA-iPD-L1 at 1452 cm^−1^ and 1438 cm^−1^. The rocking C = S vibration in the new thiourea bond was observed at 746 cm^−1^ in NOTA-iPD-L1, while the C = S vibration from isothiocyanate (NOTA–benzene–SCN) (Figure 3c) was located at 775 cm^−1^, also suggesting the correct complex synthesis.

**Figure 4:**
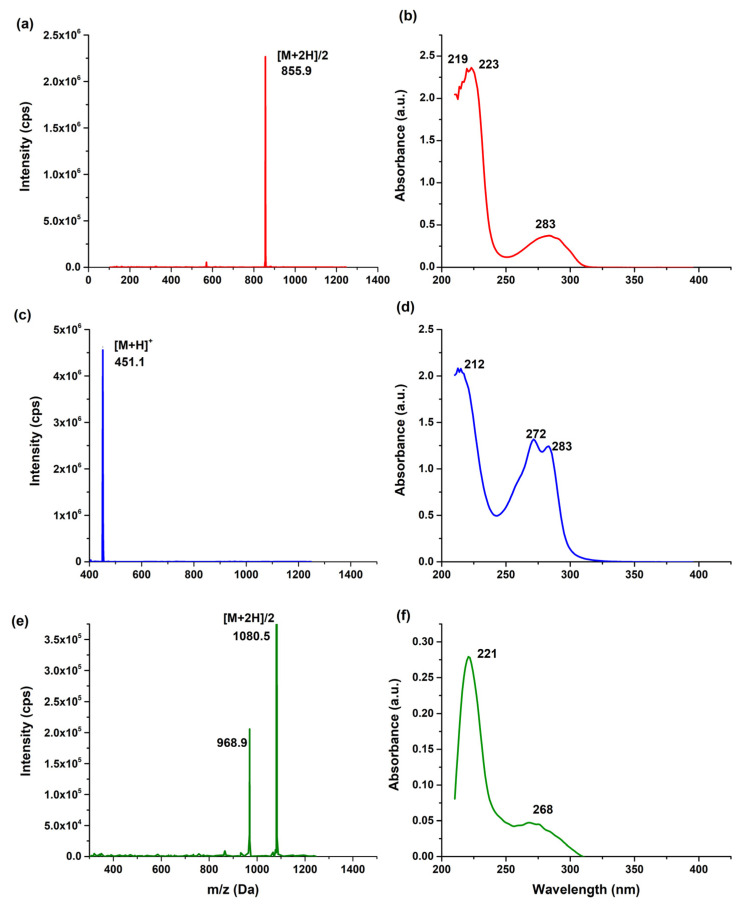
Mass spectra of (**a**) iPD-L1, (**c**) NOTA-Benzene-SCN, (**e**) NOTA-iPD-L1, and UV-Vis spectra of (**b**) iPD-L1, (**d**) NOTA–benzene–SCN, (**f**) NOTA-iPD-L1.

The mass spectra allowed for the identification of structural characteristics and fragmentation patterns. Figure 4a shows the fragment ion [M + 2]/2 [C_40_H_56_N_10_O_9_S]^2+^ from iPD-L1 at *m*/*z* 855.9 Da. Meanwhile, the NOTA–benzene–SCN spectra (Figure 4c) showed the molecular ion [M + H]+ at *m*/*z* 451.1 Da [C_20_H_28_N_4_O_6_S]^+^. It was also possible to observe the [M + 2]/2 fragment with a mass-to-charge value of 1080.5 Da [C_47_H_74_N_12_O_15_S]^2+^ of NOTA-iPD-L1 in Figure 4e. To enhance the structural characterization of iPD-L1, the absorption band at 219 nm and 223 nm were associated with the contribution of *n*→σ* transition due to the R-SH and C-N bond, which was shifted to 221 nm on the NOTA-iPD-L1 (Figure 4f) attributed to the chemical modification to form the thiourea bond between isothiocyanate of NOTA and the primary amine of iPDL-1. The absorption band centered at 283 nm, mainly attributed to the carbonyl groups in iPD-L1 (Figure 4b) and NOTA–benzene–SCN (Figure 4d), decreased in intensity in the new compound NOTA-SCN-iPD-L1. The structured absorption band at 268 nm (257–304 nm) was attributed to the interaction between carbonyl groups from iPD-L1 and NOTA–benzene–SCN.

**Figure 5:**
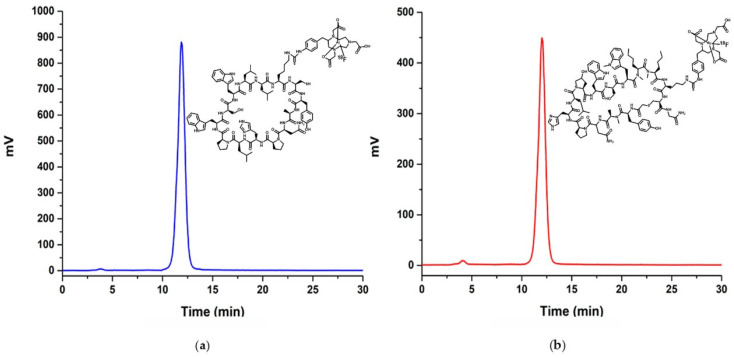
Radiochromatographic profile of (**a**) [^18^F]AlF-NOTA-iPD-L1 with t_R_ = 11.8 min, and (**b**) [^18^F]AlF-NOTA-WL12 with t_R_ = 12.0 min. Reversed phase C-18 column (L × I.D. 25 cm × 4.6 mm; particle size of 5 µm). Lineal gradient H_2_O-0.1%TFA(A)/CH_3_CN-0.1%TFA(B), from A-100% to A-30% in 20 min; flow 1 mL/min. (Insets: scheme of the radiotracer chemical structure).

The radiochemical purities, as determined by reversed-phase radio-HPLC analyses, were found to be 98.7 ± 1.2% for [^18^F]AlF-NOTA-iPD-L1 (*n* = 12) and 97.3 ± 0.7% for [^18^F]AlF-NOTA-WL12 (*n* = 12), with retention times of 11.8 ± 0.3 min and 12.04 ± 0.4 min, respectively (Figure 5). The serum stability results indicated that, after a six-hour incubation at 37 °C, the [^18^F]AlF-NOTA-iPD-L1 and [^18^F]AlF-NOTA-WL12 radiochromatograms remained unchanged, suggesting adequate radiopharmaceutical stability in the blood serum medium. The partition coefficients were determined to be 0.033 (Log D = −1.48) for [^18^F]AlF-NOTA-WL12 and 0.031 (Log D = −1.51) for [^18^F]AlF-NOTA-iPD-L1. These values indicate comparable lipophilicity between the radiopharmaceuticals, which is consistent with the retention times observed in Figure 5.

**Figure 6:**
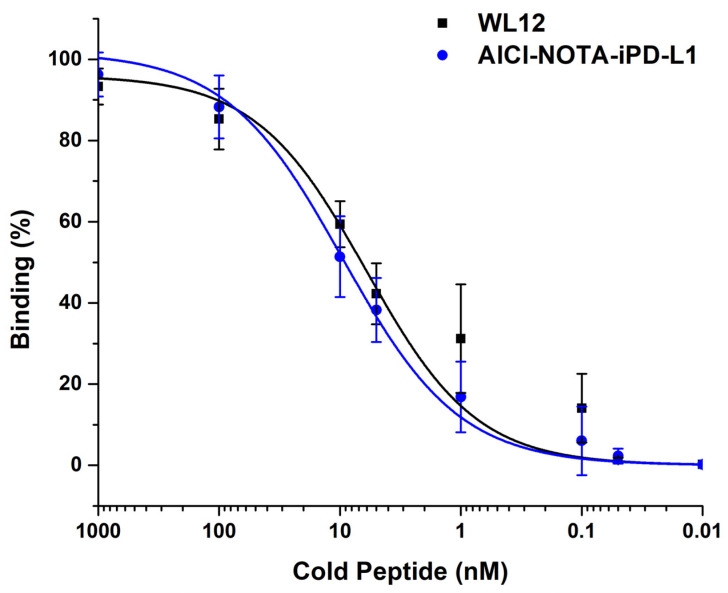
[^18^F]AlF-NOTA-iPD-L1 competition binding assay with WL12 peptide (with a proven affinity for the PD-L1 protein) and AlCl-NOTA-iPD-L1 (equivalent cold tracer of [^18^F]AlF-NOTA-iPD-L1) using 4T1 cells expressing the PD-L1 protein. No statistically significant differences were found between the IC_50_ values (Student’s *t*-test; *p* > 0.05).

The relative affinity of [^18^F]AlF-NOTA-iPD-L1 was first evaluated in competition with WL12, and then with AlCl-NOTA-iPD-L1. The latter was prepared as an approximation of the cold-formed tracer. Although molecular recognition is based on the cyclic peptide ring of iPD-L1 and not NOTA, AlCl-NOTA-iPD-L1 was generated to prevent free NOTA carboxylic acids from interfering nonspecifically with the affinity assessment and to serve as an equivalent cold tracer of [^18^F]AlF-NOTA-iPD-L1. Binding assays rely on the ability of molecules to bind to each other and form a complex that can be quantified. In this case, the IC_50_ value graphically represents the concentration of a specific ligand required to decrease the binding of a radioligand to a particular receptor by 50%. In this study, the percentage of binding of [^18^F]AlF-NOTA-iPD-L1 (with an unknown affinity for PD-L1) to 4T1 cells (PD-L1 +++) was demonstrated to be specifically displaced by different concentrations of WL12 (a standard ligand with a proven affinity for the PD-L1 protein), with an IC_50_ of 6.08 ± 1.12 nM. Likewise, the AlCl-NOTA-iPD-L1 peptide displaced the radioligand [18F]AlF-NOTA-iPD-L1 with an IC50 of 9.27 ± 2.69 nM (Figure 6). The concentration of AlCl-NOTA-iPD-L1 required to achieve a 50% reduction in this biological process is relatively comparable to several other PD-L1 inhibitors. For instance, the JBI-2174 inhibitor showed an IC50 of approximately 1 nM in a TR-FRET assay [13]. Additionally, BPI-371153 recorded an IC_50_ of less than 1 nM (by HTRF assay) [14], and the HZ-G206 demonstrated an impressively low IC_50_ value of 0.5 nM by APHAlisa in vitro assay [15]. These results clearly show that small molecules are effective PD-L1 inhibitors, and it is important to take into account the differences in molecular design and experimental conditions.

**Figure 7:**
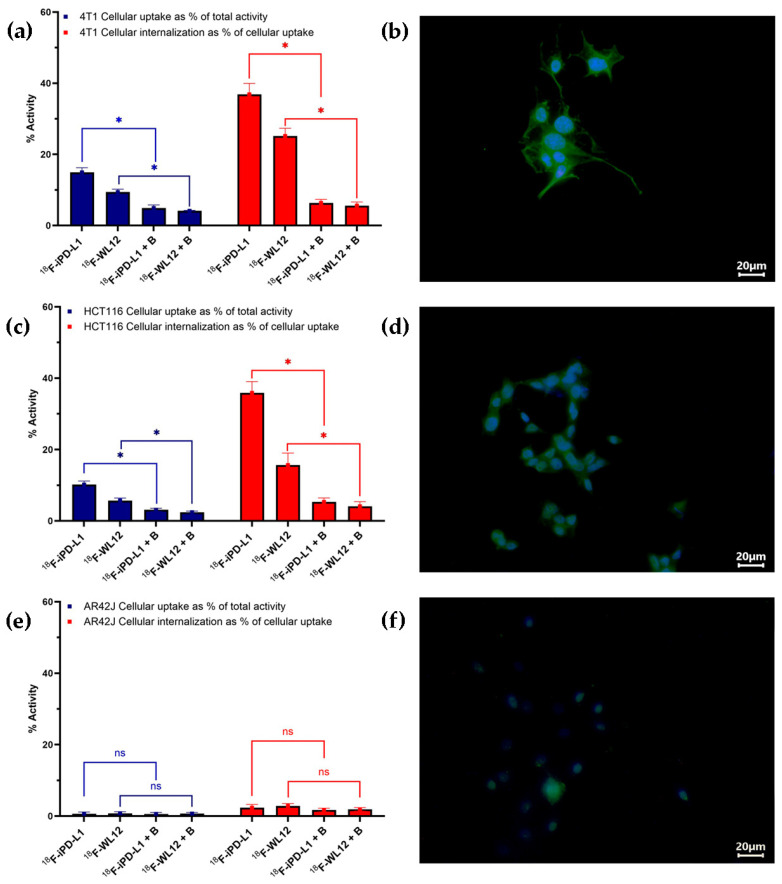
Cellular uptake and internalization of [^18^F]AlF-NOTA-iPD-L1 (^18^F-iPD-L1) and [^18^F]AlF-NOTA-WL12 (^18^F-WL12) in the (**a**) 4T1, (**c**) HCT116, and (**e**) AR42J cell lines. ns: not statisically significant. B: blocked cells (B: pre-incubation with non-labeled iPD-L1 or WL12). * Statistically significant difference (*p* < 0.05, *t*-student test). The micrographs depicted in the right panel of the figure illustrate the immunofluorescence staining for PD-L1 in (**b**) 4T1 cells (+++), (**d**) HCT116 cells (++), and (**f**) AR42J cells (low expression), respectively. The PD-L1 staining is depicted in green, while the cell nuclei are stained in blue with DAPI.

**Figure 8:**
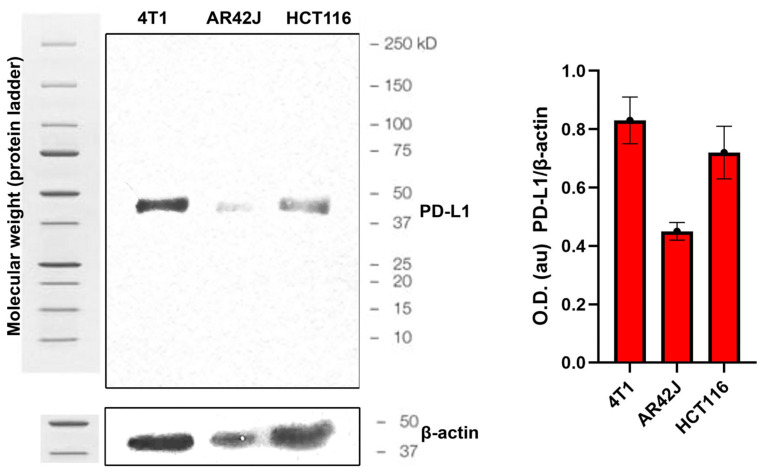
The Western blot assay demonstrated PD-L1 expression in the 4T1, AR42J, and HCT116 cell lines (see figure on the left). A graphical representation of the PD-L1/β-actin optical density (O.D.) ratio is shown in the right panel of the figure. This ratio also demonstrated low PD-L1 expression in AR42J cells. The molecular weight of PD-L1 and β-actin proteins was verified against protein ladder reference markers.

The cellular uptake and internalization of [^18^F]AlF-NOTA-iPD-L1 and [^18^F]AlF-NOTA-WL12 in 4T1, HCT116, and AR42J cell lines are shown in Figure 7. A statistically significant difference was identified (*p* < 0.05, *t*-student test) between blocked (B: pre-incubation with non-labeled iPD-L1 or WL12) and unblocked cells, suggesting a specific uptake and internalization of radiopharmaceuticals. As illustrated by the micrographs displayed in the right panels of Figure 7, PD-L1 expression was observed in 4T1 (+++), HCT116 (++), and AR42J (low expression) cells, respectively, which correlated with the observed values of [^18^F]AlF-NOTA-iPD-L1 and [^18^F]AlF-NOTA-WL12 cell uptake and internalization (Figure 5). The low levels of uptake and internalization in AR42J cells can be attributed to very low expression of PD-L1 protein on the cell surface, despite some expression in the cytoplasm (Figure 7). This phenomenon explains the presence of a certain amount of PD-L1 protein in AR42J cells observed in the Western blot assay (Figure 8). The results observed may be associated with the presence of PD-L1 in the intracellular environment, where it functions as an RNA-binding protein [8].

Biodistribution studies were conducted to evaluate the ability of [^18^F]AlF-NOTA-iPD-L1 to detect PD-L1 in vivo. Given the high level of PD-L1 expression observed in 4T1 cells, these cells were selected for the induction of murine tumors. The biodistribution of [^18^F]AlF-NOTA-iPD-L1 in mice with induced breast cancer tumors (4T1 cells) indicated a tumor uptake of 6.4 ± 0.9% ID/g at 1-h post-administration (Table 1). Three hours later, the tumor activity remained at 5.9 ± 1.1% ID/g. [^18^F]AlF-NOTA-iPD-L1 exhibits hepatobiliary (15.52% ID/g at 3 h) and renal (35.84% ID/g at 3 h) elimination (Table 1), which did not change significantly (*p* > 0.05, Student’s *t*-test) in the blocking mice group (kidney: 38.25 ± 3.21% ID/g; liver: 16.21 ± 1.98% ID/g). However, the specificity was proven by observing a significant reduction in the accumulation of [^18^F]AlF-NOTA-iPD-L1 in the tumors of mice that received a blocking dose of cold iPD-L1 peptide (100 µg) (*p* < 0.05, Student’s *t*-test) (Table 1). As previously reported [12], the formation of 4T1 triple-negative breast cancer tumors was corroborated by immunohistochemistry. Therefore, the radiopharmaceutical demonstrated potential for in vivo detection of PD-L1 expression in breast tumors.

**Figure 9:**
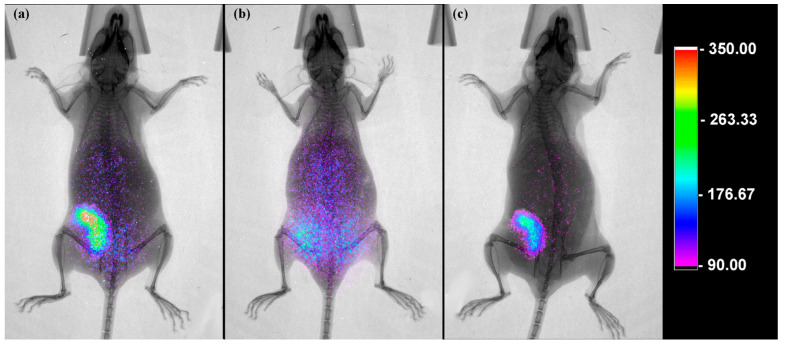
Cerenkov/X-ray images of a mouse with induced triple-negative breast cancer (4T1 tumor) acquired at (**a**) 1 h after [^18^F]AlF-NOTA-iPD-L1 administration and (**c**) 24 h after administration of [^177^Lu]Lu-DOTA-iPD-L1. (**b**) A mouse with induced triple-negative breast cancer (4T1 tumor) that received a blocking dose of non-radiolabeled iPD-L1, followed by [^18^F]AlF-NOTA-iPD-L1, with luminescent image capture at one hour after radiopharmaceutical administration.

Accumulation of both [^18^F]AlF-NOTA-iPD-L1 and [^177^Lu]Lu-DOTA-iPD-L1 was observed in the same murine breast tumor using luminescent (Cerenkov) imaging (Figure 9). Additionally, the significant reduction in [^18^F]AlF-NOTA-iPD-L1 accumulation in tumors of mice that received a blocking dose of cold iPD-L1 peptide proved the radiopharmaceutical’s specificity (Figure 9b). These results demonstrated that [^18^F]AlF-NOTA-iPD-L1 could serve as a novel radiotracer for PD-L1 PET imaging and potentially as a theranostic pair with [^177^Lu]Lu-DOTA-iPD-L1.

It is essential to note that Cerenkov molecular imaging, being a 2D technique, provides a superficial image of biological activity, whereas PET/CT, being a 3D technique, offers a volumetric representation of the same, enabling an accurate assessment of the depth distribution of metabolic activity in various organs and tissues. This is why the tumor activity of [^18^F]AlF-NOTA-iPD-L1 observed in the murine Cerenkov images shown in Figure 9 overlaps with the signal that could be detected from metabolic organs where the radiotracer is also distributed, as shown in Table 1. However, although both [^18^F]AlF-NOTA-iPD-L1 and [^177^Lu]Lu-DOTA-iPD-L1 accumulated in the same murine breast tumor, as demonstrated by Cerenkov imaging (proof-of-concept), PET/CT imaging is necessary to confirm these results and to confirm the potential of these radiopharmaceuticals as a promising theranostic pair.

## 4. Discussion

PD-L1 molecular imaging is a critical tool in the field of cancer immunotherapy, offering significant benefits, including non-invasive, real-time assessment. Dynamic visualization of PD-L1 expression enables continuous monitoring of tumor response to therapy, which is essential for patient stratification and evaluating the effectiveness of immunotherapy. Additionally, molecular imaging enables the accurate selection of patients who are more likely to benefit from PD-1/PD-L1 inhibitors by providing insights into the tumor microenvironment and interactions between the tumor and the immune system. These insights help tailor treatment plans to individual patients, improving treatment outcomes [16,17,18]. However, most radiotracers based on small molecules targeting the PD-L1 protein for PET imaging are designed for labeling with ^68^Ga (e.g., [^68^Ga]Ga-NODAGA-WL12 [^68^Ga]Ga-DOTA-HN11-1, [^68^Ga]Ga-DOTA-NK224, [^68^Ga]Ga-DOTA-HF12, [^68^Ga]Ga-DOTA-CCC, [^68^Ga]Ga-DOTA-PG1) [17,18,19,20,21,22] and, to a lesser extent, with ^18^F. It has been established that the lower positron energy of ^18^F (Emax = 633.5 keV, 96.7%) in comparison to ^68^Ga (E_max_ = 1899 keV, 87.7%) results in a reduced mean positron range (0.56 mm vs. 3.5 mm). This phenomenon leads to enhanced PET image quality, higher resolution, and improved diagnostic quality [23]. For example, a direct comparative study of PET/CT image quality and spatial resolution obtained with ^18^F and ^68^Ga revealed that ^18^F yielded superior image quality due to the significantly higher energy of the positrons emitted from the decay of ^68^Ga, which degrades image quality and spatial resolution [24].

[^18^F]AlF-NOTA-iPD-L1 was obtained with a radiochemical purity greater than 97%, and it demonstrated high in vitro and in vivo stability, and specific recognition by the PD-L1 protein. [^68^Ga]Ga-radiotracers evaluated for imaging PD-L1 expression in tumors have demonstrated high binding affinities, with IC_50_ values ranging from 2.45 to 113.2 nM, and specific tumor uptake values from 3.36 to 11.56 ID/g [17,18,19,20,21,22]. These findings highlight the potential of these radiotracers for non-invasive imaging and quantification of PD-L1 levels in tumors. In this study, AlCl-NOTA-iPD-L1 showed an IC_50_ of 9.27 ± 2.69 nM and a 4T1 tumor uptake (PD-L1 +++) of [^18^F]AlF-NOTA-iPD-L1 of 6.4% ± 0.9% ID/g, confirming its effectiveness and specificity in detecting PD-L1 protein.

Recent developments in the field of PET radiotracers have led to the creation of several ^18^F-peptide molecules targeting the PD-L1 protein, such as [^18^F]AlF-BCY10959, [^18^F]LG-2/LG-3, and [^18^F]AlF-NOTA-PCP2 [25,26,27], which also exhibit equivalent molecular recognition properties to those of [^18^F]AlF-NOTA-iPD-L1. For instance, the bicyclic peptide-based radiotracers [^18^F]AlF-BCY509 and [^18^F]AlF-BCY10959 exhibited high specificity and affinity, with IC_50_ values of 9.36 ± 1.35 and 7.12 ± 1.24 nM, respectively, and Kd values of 11.41 ± 1.04 and 8.09 ± 0.85 nM, respectively [25]. In PET imaging, [^18^F]AlF-BCY10959 showed significant accumulation in PD-L1-positive tumors, exhibiting a tumor uptake of 14.74% ± 1.67% ID/mL [25]. Similarly, PET imaging in mice bearing B16-F10 tumors displayed tumor uptake of 6.45% ± 0.38% ID/mL for [18F]LG-2 and 5.64% ± 0.02% ID/mL for [18F]LG-3 [26]. Additionally, [18F]AlF-NOTA-PCP2 exhibited high-affinity binding to PD-L1, with a Kd value of 0.24 nM. PET/CT imaging revealed significant accumulation of [18F]AlF-NOTA-PCP2 in orthotopic tumors (9.51% ± 0.73% ID/mL) [27]. Therefore, these radiotracers are promising options for detecting PD-L1 expression and monitoring its dynamics during treatment. Despite varying degrees of tumor uptake evaluated by PET imaging (%ID/mL) and binding affinities, the results for [^18^F]AlF-NOTA-iPD-L1 fall within the range of those shown by these other ^18^F-radiotracers. However, to the best of our knowledge, no targeted radiotherapeutic pair has been reported for any of these PD-L1 inhibitors labeled with ^18^F or those labeled with ^68^Ga. Therefore, it is essential to note that the aim of this research was not to compare the preclinical data of [^18^F]AlF-NOTA-iPD-L1 with those of various radiotracers targeting PD-L1, but rather to show that [^18^F]AlF-NOTA-iPD-L1 can be helpful as a theranostic pair with [^177^Lu]Lu-DOTA-iPD-L1; paving the way for advancements in cancer diagnosis and theranostics with focus on the immunomodulatory effect of the ^177^Lu radiopharmaceutical pair [12], moving beyond its simple tumor radiation absorbed dose.

It has been demonstrated that the radiation emitted by ^177^Lu stimulates a local inflammatory reaction, specifically in the tumor where the radiopharmaceutical selectively accumulates. This increase in inflammation leads to enhanced penetration of tumor-specific T cells into the tumor microenvironment, accompanied by a concurrent increase in the expression of PD-L1 in cancer cells. Consequently, the concept of PD-L1 expression induced by [^177^Lu]Lu-DOTA-iPD-L1 (radiation) and the subsequent blockade by the administration of anti-PD-L1/PD-1 or PD-L1 inhibitor agents may represent a potent therapeutic modality, particularly in the context of triple-negative breast cancer [12]. Furthermore, the augmentation of infiltrated active macrophages, consequent to the effect of ^177^Lu radiation, contributes to the production of molecules that inhibit tumor cell growth, blood vessel formation, cell adhesion, and tissue architecture [10,12].

[^18^F]AlF-NOTA-iPD-L1 demonstrated a comparable biodistribution profile to [^177^Lu]Lu-DOTA-iPD-L1 [12]. Based on the tumor uptake (6.97 ± 1.04% ID at 1 h and 5.74 ± 1.01% ID at 24 h) and hepatobiliary and renal clearance, it has been calculated that [^177^Lu]Lu-DOTA-iPD-L1 delivers a tumor dose of 27 Gy/37 MBq and less than 0.36 Gy/37 MBq to non-target organs [12]. Thus, despite the short half-life of [^18^F]AlF-NOTA-iPD-L1, it can be useful for initially determining tumor PD-L1 expression, and thereby the feasibility of administering [^177^Lu]Lu-DOTA-iPD-L1 as a combined modality of targeted radiotherapy and anti-PD-L1 immunotherapy.

A relevant point regarding the development of [^18^F]AlF-NOTA-iPD-L1 and other radiotracers based on PD-L1 inhibitor molecules is their potential to target PD-L1 expression precisely throughout the tumor. This overcomes the problem of tumor heterogeneity, which affects biopsy results when PD-L1 expression is evaluated only in a small sample of tumor tissue [16].

Given the significance of the immunomodulatory effect of [^177^Lu]Lu-DOTA-iPD-L1, the potential impact of [^18^F]AlF-DOTA-iPD-L1 developed in this research is particularly relevant for the selection, monitoring, and enhancement of outcomes in cancer patients undergoing concurrent anti-PD-L1/PD-1 immunotherapy. The development of the first reported radiotheranostic pair targeting PD-L1 (^18^F-iPD-L1/[^177^Lu]Lu-DOTA-iPD-L1), which utilizes the same molecular iPD-L1 probe for both targeted ^177^Lu-radiotherapy and diagnostic imaging by ^18^F-PET, may help facilitate the completion of this endeavor.

## 5. Conclusions

PD-L1 molecular imaging significantly enhances cancer diagnosis and treatment by providing a non-invasive, real-time, and comprehensive assessment of PD-L1 expression. This leads to better patient stratification, monitoring of therapeutic efficacy, and the development of theranostic agents, ultimately improving clinical outcomes for cancer patients. It has previously been reported that [^177^Lu]Lu-DOTA-iPD-L1 targeting the programmed cell death ligand-1 in tumors may potentially promote immune responses [12], which could support a shift towards more tailored and effective immunotherapy strategies. [^18^F]AlF-NOTA-iPD-L1, developed in this research, demonstrated high in vitro and in vivo stability, specific recognition by the PD-L1 protein, and adequate properties to function as a theranostic pair for [^177^Lu]Lu-DOTA-iPD-L1. Therefore, these results warrant further studies to evaluate the clinical usefulness of [^18^F]AlF-NOTA-iPD-L1/^177^Lu-iPD-L1 as a radiotheranostic pair.

## Figures and Tables

**Table 1:** Biodistribution of [^18^F]AlF-NOTA-iPD-L1 in mice with induced PD-L1-positive 4T1 tumors (% ID/g) (*n* = 3).

Organ/Tissue	Time (h)	% ID/g
Kidney	1 2 3	47.43 ± 5.38 52.31 ± 4.73 35.84 ± 3.91
Liver	1 2 3	22.66 ± 3.13 19.15 ± 3.34 15.52 ± 2.38
Heart	1 2 3	1.62 ± 0.47 0.91 ± 0.55 0.47 ± 0.11
Spleen	1 2 3	1.69 ± 0.24 1.26 ± 0.25 0.84 ± 0.13
Lung	1 2 3	1.81 ± 0.35 0.78 ± 0.23 0.37 ± 0.11
Intestine	1 2 3	0.22 ± 0.17 0.61 ± 0.35 0.83 ± 0.37
Unblocking tumor (4T1)	1 2 3	6.49 ± 0.98 * 6.54 ± 1.23 5.97 ± 1.31
Blocking tumor (4T1)	1	1.81 ± 1.03 *

* Statistically significant difference (*p* < 0.05, *t*-student).

## Data Availability

Data is contained within the article.
